# Genetic Diversity of Autosomal STR Markers in the Brahmin Population of Rajasthan and Haryana: Significance in Population and Forensic Genetics

**DOI:** 10.17691/stm2023.15.1.07

**Published:** 2023-01-28

**Authors:** Shivkant Sharma, Vivek Sahajpal, Abhishek Singh, Ritu Yadav, Mukesh Thakur, Deepika Bhandari, Shalu Ranga, Lokesh Kadian, Chetna Yadav

**Affiliations:** Research Scholar; Department of Genetics, Maharshi Dayanand University, Rohtak, Haryana, 124001, India;; Assistant Director; Directorate of Forensics Services, Himachal Pradesh, Junga-171218, India;; Research Scholar; Zoological Survey of India, New Alipore, Kolkata, 700053, India;; Associate Professor; Department of Genetics, Maharshi Dayanand University, Rohtak, Haryana, 124001, India;; Scientist C; Zoological Survey of India, New Alipore, Kolkata, 700053, India;; Assistant Professor; Government Institute of Forensic Science, Mumbai, Maharshtra, 400032, India; Research Scholar; Department of Genetics, Maharshi Dayanand University, Rohtak, Haryana, 124001, India;; Research Scholar; Department of Genetics, Maharshi Dayanand University, Rohtak, Haryana, 124001, India;; Research Scholar; Department of Genetics, Maharshi Dayanand University, Rohtak, Haryana, 124001, India;

**Keywords:** genetic diversity, population genetics, Brahmin population, Rajasthan, Haryana

## Abstract

**The aim of the study** is to evaluate the suitability of STRs for molecular characterization and forensic applications in unrelated Brahmins of Rajasthan and Haryana states, India.

**Materials and Methods:**

A total of 203 male DNA samples from various districts of Haryana (n=104) and Rajasthan (n=99) were genotyped using the GlobalFiler^®^ PCR Amplification Kit. Allelic frequencies and different forensic parameters like PD, PE, PIC, PM, Ho, He, UHe, and TPI were calculated with different software.

**Results:**

More than 200 alleles were present in both populations, ranging from 6.0 to 35.2 and SE33 was the most polymorphic marker. The combined power of discrimination was 1. To know the relatedness with other Indian Brahmin populations, the UPGMA dendrogram and principal component analysis plot were visualized to show that both populations are close to each other and in nearby Saraswat Brahmins of Himachal Pradesh. This study showed a genetic relationship and forensic examination in the Haryana and Rajasthan Brahmin populations and various ethno-linguistically diverse populations of India.

**Conclusion:**

The results imply that the highly polymorphic 21 autosomal STR loci might be applied for individuals’ forensic identification and parentage testing. This study also suggests that the kit having both autosomal and Y-STR markers is appropriate for a better understanding of the genetic and forensic examination in the Brahmin population of Haryana and Rajasthan.

## Introduction

Genetic diversity is essential for the evolution of a species, and it offers the genetic make-up of a species based on the total number of gene distinctiveness features. Diversity reasonably determines population or human social organization in language, ethnicity, culture, geography, social aspect, and communities worldwide. These characteristics are common in the alpine environment, where people frequently live in separate communities far from developed areas [1].

Some traditions emerged due to the assimilation of varied cultures and others died over a period of time. On the other hand, certain cultures have remained steadfast and persistent in demonstrating their presence over time [2]. India has always welcomed people of many racial groups and ethnicities. According to various pieces of evidence, it is possible that modern people left Africa via the southern coastal route and India emerged as a vital human mobility corridor by the initial wave of migration along the south coast route [3]. The interaction and conversion of ideas may have resulted in hybridization between populations of various ethnolinguistic categories in India that further resulted in cultural, linguistic, and genetic variation [4]. In 1919, Ludwik and Hanka Hirszfeld, pioneers in blood typing, first reported the genetic variation among human populations [5]. However, the variations studied in blood groups were insufficient to identify a particular individual from a gene pool. Thus, this area of interest was revolutionized by detecting variations in the human gene pool at the DNA level [6]. In the early days, restriction fragment length polymorphism analysis was employed for DNA analysis. Later on PCR-based assays (with increased sensitivity and power of discrimination) were used along with SNPs, VNTRs, and STRs. In 1990, Alec Jeffrey, with the Human Genome Project, created a wide range of STR sequences [7].

By the mid-1990s, multiple STR markers were added in single multiplexed reactions in forensic DNA testing. Several studies have been used with autosomal STR markers to investigate genetic relationships among Indian groups. The caste system in India is supported by four primary groups or varna: Brahmin, Kshatriya, Vaishya, and Sudra. The castes are further divided into sub-castes and individuals belonging to these sub-castes practice endogamy, a system in which an individual marries inside a specific group [8].

**The aim of the study** was to investigate the molecular characterization and forensic applications of Brahmins who practice endogamy i.e. they marry within the same caste, practice gotras (clan) system, and do not marry in the same gotras.

## Materials and Methods

### DNA isolation and ethical consideration

Present study was conducted on stored DNA samples in lab G-22, Department of Genetics, Maharshi Dayanand University (Rohtak, Haryana, India) [9]. A total of 203 male DNA samples from various districts of Haryana (n=104) and Rajasthan (n=99) were used. For autosomal STR analysis in Haryana and Rajasthan Brahmin population, ethical clearance was taken from the Institutional Human Ethical Committee (IHEC) vide letter No.IHEC/2021/289 dated September 9, 2021.

### PCR amplification and genotyping

1.2-mm punch of the FTA card containing the DNA samples was used for amplification [10]. Twenty-one autosomal and three sex-determining markers were amplified in a total volume of 25 μl using the GlobalFiler^®^ PCR Amplification Kit (Thermo Fisher Scientific, USA) according to the manufacturer’s instructions. Throughout the reactions, positive and negative controls were employed. Capillary electrophoresis was used to separate and detect amplified products using an ABI 3500xL Genetic Analyzer (Applied Biosystem, USA). GeneMapper IDX version 1.6 (Applied Biosystem, USA) was utilized for manual allele calling.

### Statistical data processing

To calculate the allelic frequency distribution, as well as forensic parameters like polymorphism information content (PIC), probability of match (PM), typical paternity index (TPI), observed heterozygosity (Ho), expected heterozygosity (He), and unbiased heterozygosity (UHe) for 21 autosomal STR loci, STRAF (STR analysis for forensics) online software [11] and GenAlEx 6.5 were used [12]. Additionally, a UPGMA dendrogram [13] was generated based on the D_sw_ distance determined by POPTREE2 software [14] to examine the genetic distance between the present study population and formerly observed 10 Brahmin groups of India. The principal component analysis (PCA) plot was also created with the help of allelic frequency data through Paleontological Statistics (PAST) software version 3.02 [15] to evaluate the grouping pattern between these populations.

### Quality assurance

A positive control DNA template (2800 M), provided along with the kit, and negative controls were used throughout the study. The experimental work was carried out in an accredited laboratory conforming to ISO/IEC 17025 standard. Further, the authors have also qualified International DNA Proficiency Test (http://gitad.ugr.es/principal.htm).

## Results and Discussion

In the Haryana Brahmin population, a total of 61 distinct alleles were detected in the Haryana Brahmin community, ranging from 6.0 to 34.2 and 229 alleles with an average of 10.409. Table 1 shows the allele frequency distribution among Haryana Brahmins. The observed allele frequency ranged from 0.005 to 0.452, showing allele 11 (0.452) as the most common in the study population at the D2S441 locus. The researchers discovered Ho as 0.760±0.040, He as 0.789±0.022, and UHe as 0.793±0.022. Table 2 lists the forensic parameters that were evaluated, including PIC, power of exclusion (PE), power of discrimination (PD), PM and TPI as well as number of alleles (Na), number of effective alleles (Ne), Ho, UHe, and He. With 0.940 (PIC) and 0.986 (PD), locus SE33 was the most polymorphic and discriminative. Moreover, in all 21 autosomal STRs, PD, PE, PIC, PM, Ho, He, and UHe ranged from 0.851 (D2S441) to 0.986 (SE33), 0.347 (D2S441) to 0.843 (D21S11), 0.640 (D2S441) to 0.940 (SE33), 0.030 (D1S1656) to 0.149 (D2S441), 0.644 (D2S441) to 0.923 (D21S11), 0.688 (D2S441) to 0.943 (SE33), and 0.692 (TPOX) to 0.948 (SE33), respectively. The combined power of discrimination and exclusion were 1.0 and 0.823, respectively. As all the samples were confirmed males, 3 out of 104 samples showed the amelogenin Y deletion, i.e. they show only X allele at amelogenin marker. All these samples were also confirmed by the Y-STR profiling.

**Table 1:** Observed allelic frequency distribution in the Haryana Brahmin population at 21 autosomal STRs

Allele	CSF1PO	D10S1248	D12S391	D13S317	D16S539	D18S51	D19S433	D1S1656	D21S11	D22S1045	D2S1338	D2S441	D3S1358	D5S818	D7S820	D8S1179	DYS391	FGA	SE33	TH01	TPOX
6	—	—	—	—	—	—	—	—	—	—	—	—	—	—	—	—	—	—	—	0.264	—
7	—	—	—	0.005	—	—	—	—	—	—	—	—	—	0.014	0.029	—	—	—	—	0.159	—
8	—	—	—	0.178	0.058	—	0	0.024	—	—	—	—	—	—	0.183	0.005	—	—	—	0.12	0.375
9	0.043	—	—	0.101	0.13	—	—	0.005	—	—	—	0	—	0.048	0.087	0.005	0	—	—	0.264	0.125
9.3	—	—	—	—	—	—	—	—	—	—	—	—	—	—	0.005	—	—	—	—	0.183	—
10	0.245	—	—	0.082	0.159	0.01	—	0.005	0	0.005	—	0.288	0.005	0.063	0.168	0.163	0.702	—	0	0.01	0.106
11	0.308	0.005	—	0.288	0.308	0.01	—	0.135	—	0.24	—	0.452	—	0.303	0.308	0.067	0.288	—	—	—	0.346
11.3	—	—	—	—	—	—	—	—	—	—	—	0.019	—	—	—	—	—	—	—	—	—
11.5	—	—	—	—	—	—	—	0.005	—	—	—	—	—	—	—	—	—	—	—	—	—
12	0.308	0.005	0.005	0.245	0.188	0.087	0.101	0.12	—	0.005	—	0.063	—	0.337	0.183	0.087	0.01	—	0	—	0.043
12.2	—	—	—	—	—	—	0.005	—	—	—	—	—	—	—	—	—	—	—	—	—	—
12.3	—	—	—	—	—	—	—	—	—	—	—	0	—	—	—	—	—	—	—	—	—
13	0.067	0.12	0	0.077	0.13	0.082	0.284	0.144	—	0.01	0.005	0.019	—	0.221	0.034	0.188	—	—	0.005	—	0.005
13.2	—	—	—	—	—	—	0.019	—	—	—	—	—	—	—	—	—	—	—	—	—	—
14	0.029	0.303	—	0.024	0.029	0.274	0.24	0.077	—	0.087	—	0.139	0.053	0.014	0.005	0.25	—	—	0.019	—	—
14.2	—	—	—	—	—	—	0.063	—	—	—	—	—	—	—	—	—	—	—	—	—	—
15	—	0.274	0.01	—	—	0.144	0.135	0.135	—	0.385	—	0.01	0.385	—	—	0.139	—	—	0.019	—	—
15.2	—	—	—	—	—	—	0.048	—	—	—	—	—	—	—	—	—	—	—	—	—	—
15.3	—	—	—	—	—	—	—	0.048	—	—	—	—	—	—	—	—	—	—	—	—	—
16	—	0.212	0.01	—	—	0.144	0.058	0.159	—	0.173	0.005	0.01	0.226	—	—	0.067	—	—	0.053	—	—
16.2	—	—	—	—	—	—	0.034	—	—	—	—	—	—	—	—	—	—	—	0.005	—	—
16.3	—	—	—	—	—	—	—	0.024	—	—	—	—	—	—	—	—	—	—	—	—	—
17	—	0.077	0.111	—	—	0.072	0.014	0.043	—	0.082	0.053	—	0.212	—	—	0.029	—	—	0.063	—	—
17.2	—	—	—	—	—	—	0	—	—	—	—	—	—	—	—	—	—	—	—	—	—
17.3	—	—	0.005	—	—	—	—	0.043	—	—	—	—	—	—	—	—	—	—	—	—	—
18	—	0.005	0.279	—	—	0.072	—	0.014	—	0.014	0.139	—	0.115	—	—	—	—	0.019	0.115	—	—
18.3	—	—	0.01	—	—	—	—	0.01	—	—	—	—	—	—	—	—	—	—	—	—	—
19	—	—	0.12	—	—	0.082	—	—	—	—	0.168	—	0	—	—	—	—	0.048	0.072	—	—
19.2	—	—	—	—	—	—	—	—	—	—	—	—	—	—	—	—	—	—	0.005	—	—
19.3	—	—	0.005	—	—	—	—	0.01	—	—	—	—	—	—	—	—	—	—	—	—	—
20	—	—	0.12	—	—	0.014	—	—	—	—	0.154	—	0.005	—	—	—	—	0.139	0.096	—	—
20.2	—	—	—	—	—	—	—	—	—	—	—	—	—	—	—	—	—	—	0.024	—	—
21	—	—	0.096	—	—	0.01	—	—	—	—	0.024	—	—	—	—	—	—	0.135	0.019	—	—
21.1	—	—	—	—	—	—	—	—	—	—	—	—	—	—	—	—	—	—	0.01	—	—
21.2	—	—	—	—	—	—	—	—	—	—	—	—	—	—	—	—	—	—	0.01	—	—
21.3	—	—	—	—	—	—	—	—	—	—	—	—	—	—	—	—	—	0.005	—	—	—
22	—	—	0.087	—	—	—	—	—	—	—	0.101	—	—	—	—	—	—	0.149	0.019	—	0
22.2	—	—	0	—	—	—	—	—	—	—	—	—	—	—	—	—	—	0.005	0.014	—	—
23	—	—	0.087	—	—	—	—	—	—	—	0.139	—	—	—	—	—	—	0.154	0.005	—	—
23.2	—	—	—	—	—	—	—	—	—	—	—	—	—	—	—	—	—	0	0.019	—	—
24	—	—	0.038	—	—	—	—	—	—	—	0.135	—	—	—	—	—	—	0.159	0.005	—	—
24.2	—	—	—	—	—	—	—	—	—	—	—	—	—	—	—	—	—	0.014	0.014	—	—
25	—	—	0.019	—	—	—	—	—	—	—	0.048	—	—	—	—	—	—	0.13	—	—	—
25.2	—	—	—	—	—	—	—	—	—	—	—	—	—	—	—	—	—	—	0.038	—	—
26	—	—	—	—	—	—	—	—	—	—	0.029	—	—	—	—	—	—	0.038	—	—	—
26.2	—	—	—	—	—	—	—	—	—	—	—	—	—	—	—	—	—	—	0.034	—	—
27	—	—	—	—	—	—	—	—	0.029	—	—	—	—	—	—	—	—	0.005	—	—	—
27.2	—	—	—	—	—	—	—	—	—	—	—	—	—	—	—	—	—	—	0.043	—	—
28	—	—	—	—	—	—	—	—	0.154	—	0	—	—	—	—	—	—	0	—	—	—
28.2	—	—	—	—	—	—	—	—	—	—	—	—	—	—	—	—	—	—	0.058	—	—
29	—	—	—	—	—	—	—	—	0.207	—	—	—	—	—	—	—	—	—	—	—	—
29.2	—	—	—	—	—	—	—	—	—	—	—	—	—	—	—	—	—	—	0.067	—	—
30	—	—	—	—	—	—	—	—	0.178	—	—	—	—	—	—	—	—	—	0	—	—
30.2	—	—	—	—	—	—	—	—	0.024	—	—	—	—	—	—	—	—	—	0.058	—	—
31	—	—	—	—	—	—	—	—	0.019	—	—	—	—	—	—	—	—	—	—	—	—
31.2	—	—	—	—	—	—	—	—	0.125	—	—	—	—	—	—	—	—	—	0.043	—	—
32	—	—	—	—	—	—	—	—	0.005	—	—	—	—	—	—	—	—	—	—	—	—
32.2	—	—	—	—	—	—	—	—	0.183	—	—	—	—	—	—	—	—	—	0.034	—	—
33	—	—	—	—	—	—	—	—	—	—	—	—	—	—	—	—	—	—	0.019	—	—
33.2	—	—	—	—	—	—	—	—	0.067	—	—	—	—	—	—	—	—	—	0.01	—	—
34.2	—	—	—	—	—	—	—	—	0.005	—	—	—	—	—	—	—	—	—	0.005	—	—

**Table 2:** Different forensic parameters in the Brahmin population of Haryana

Locus	PIC	PM	PD	PE	TPI	Na	Ne	Ho	He	uHe
CSF1PO	0.699	0.108	0.892	0.477	1.857	7.000	3.940	0.740	0.746	0.750
D10S1248	0.730	0.092	0.908	0.462	1.793	8.000	4.310	0.721	0.768	0.772
D12S391	0.840	0.042	0.958	0.650	2.889	15.000	6.883	0.827	0.855	0.859
D13S317	0.774	0.080	0.920	0.632	2.737	8.000	5.041	0.817	0.802	0.806
D16S539	0.781	0.066	0.934	0.526	2.080	7.000	5.185	0.760	0.807	0.811
D18S51	0.836	0.046	0.954	0.650	2.889	12.000	6.737	0.827	0.852	0.856
D19S433	0.801	0.054	0.946	0.613	2.600	11.000	5.623	0.808	0.822	0.826
D1S1656	0.880	0.030	0.970	0.745	4.000	18.000	9.182	0.885	0.891	0.895
D21S11	0.828	0.054	0.946	0.843	6.500	12.000	6.518	0.923	0.847	0.851
D22S1045	0.713	0.104	0.896	0.477	1.857	9.000	3.997	0.731	0.750	0.753
D2S1338	0.861	0.037	0.963	0.745	4.000	12.000	7.953	0.875	0.874	0.878
D2S441	0.640	0.149	0.851	0.347	1.405	8.000	3.208	0.644	0.688	0.692
D3S1358	0.699	0.111	0.889	0.493	1.926	7.000	3.848	0.740	0.740	0.744
D5S818	0.695	0.125	0.875	0.632	2.737	7.000	3.838	0.817	0.739	0.743
D7S820	0.773	0.082	0.918	0.650	2.889	9.000	5.019	0.827	0.801	0.805
D8S1179	0.819	0.054	0.946	0.578	2.364	10.000	6.202	0.788	0.839	0.843
DYS391	0.342	0.576	0.424	0.000	0.500	3.000	1.736	0.000	0.424	0.426
FGA	0.856	0.039	0.961	0.803	5.200	13.000	7.698	0.904	0.870	0.874
SE33	0.940	0.014	0.986	0.823	5.778	31.000	17.616	0.913	0.943	0.948
TH01	0.753	0.078	0.922	0.431	1.677	6.000	4.696	0.702	0.787	0.791
TPOX	0.662	0.143	0.857	0.462	1.793	7.000	3.501	0.731	0.714	0.718
vWA	0.776	0.067	0.933	0.493	1.926	9.000	5.095	0.740	0.804	0.808
Mean		7.82E–26	1.000	0.999	3.5E+08	10.409	5.810	0.760	0.789	0.793
SE						1.207	0.675	0.040	0.022	0.022

In the Rajasthan Brahmin population, 62 distinct alleles were detected, ranging from 6.0 to 35.2 and a total of 222 alleles with an average of 10.091 alleles

per locus were discovered. Table 3 shows the allele frequency distribution among Rajasthan Brahmins. The observed allele frequency ranged from 0.005 to 0.409, showing allele 8 (0.409) as the most common in the examined population and the TPOX locus having the highest allele frequency. It was discovered that the values of Ho as 0.771±0.039, He as 0.787±0.022, and UHe as 0.791±0.022 were present. Table 4 shows the evaluated forensic parameters such as PIC, PD, PE, TPI, and PM, as well as Na, Ne, Ho, He, and UHe. With 0.935 (PIC) and 0.982 (PD), locus SE33 was discovered to be the most polymorphic and discriminative. Moreover, in all 21 autosomal STRs, PD, PE, PIC, PM, Ho, He, and UHe were found ranged from 0.850 (CSF1PO) to 0.982 (SE33), 0.408 (TPOX) to 0.814 (D18S51), 0.648 (TPOX) to 0.935 (SE33), 0.029 (D1S1656) to 0.150 (CSF1PO), 0.687 (TPOX) to 0.909 (D18S51), 0.698 (TPOX) to 0.938 (SE33), and 0.702 (TPOX) to 0.943 (SE33), respectively. The CPD was noted 1 and CPE was 0.999. In this population, only one male out of 100 showed the amelogenin Y deletion [16].

**Table 3:** Observed allelic frequency distribution in the Rajasthan Brahmin population at 21 autosomal STRs

Allele	CSF1PO	D10S1248	D12S391	D13S317	D16S539	D18S51	D19S433	D1S1656	D21S11	D22S1045	D2S1338	D2S441	D3S1358	D5S818	D7S820	D8S1179	DYS391	FGA	SE33	TH01	TPOX
6	—	—	—	—	—	—	—	—	—	—	—	—	—	—	—	—	—	—	—	0.242	—
7	—	—	—	0.020	—	—	—	—	—	—	—	—	—	0.000	0.020	—	—	—	—	0.187	—
8	—	—	—	0.152	0.071	—	0.005	0.035	—	—	—	—	—	—	0.222	0.025	—	—	—	0.131	0.409
9	0.025	—	—	0.071	0.131	—	—	0.015	—	—	—	0.005	—	0.025	0.091	0.010	0.030	—	—	0.253	0.116
9.3	—	—	—	—	—	—	—	—	—	—	—	—	—	—	0.000	—	—	—	—	0.177	—
10	0.202	—	—	0.071	0.101	0.000	—	0.010	0.010	0.005	—	0.247	0.000	0.116	0.172	0.111	0.707	—	0.005	0.010	0.086
11	0.293	0.010	—	0.273	0.338	0.020	—	0.172	—	0.202	—	0.404	—	0.323	0.253	0.081	0.253	—	—	—	0.333
11.3	—	—	—	—	—	—	—	—	—	—	—	0.030	—	—	—	—	—	—	—	—	—
11.5	—	—	—	—	—	—	—	0.000	—	—	—	—	—	—	—	—	—	—	—	—	—
12	0.404	0.015	0.000	0.338	0.177	0.101	0.076	0.086	—	0.005	—	0.056	—	0.364	0.202	0.126	0.010	—	0.015	—	0.051
12.2	—	—	—	—	—	—	0.010	—	—	—	—	—	—	—	—	—	—	—	—	—	—
12.3	—	—	—	—	—	—	—	—	—	—	—	0.005	—	—	—	—	—	—	—	—	—
13	0.061	0.157	0.005	0.061	0.162	0.146	0.364	0.121	—	0.020	0.000	0.035	—	0.162	0.035	0.207	—	—	0.005	—	0.000
13.2	—	—	—	—	—	—	0.035	—	—	—	—	—	—	—	—	—	—	—	—	—	—
14	0.015	0.212	—	0.015	0.020	0.217	0.217	0.081	—	0.061	—	0.152	0.040	0.010	0.005	0.212	—	—	0.030	—	—
14.2	—	—	—	—	—	—	0.056	—	—	—	—	—	—	—	—	—	—	—	—	—	—
15	—	0.338	0.005	—	—	0.172	0.101	0.136	—	0.460	—	0.051	0.308	—	—	0.172	—	—	0.015	—	—
15.2	—	—	—	—	—	—	0.056	—	—	—	—	—	—	—	—	—	—	—	—	—	—
15.3	—	—	—	—	—	—	—	0.015	—	—	—	—	—	—	—	—	—	—	—	—	—
16	—	0.202	0.010	—	—	0.101	0.051	0.116	—	0.162	0.005	0.015	0.273	—	—	0.035	—	—	0.051	—	—
16.2	—	—	—	—	—	—	0.015	—	—	—	—	—	—	—	—	—	—	—	0.000	—	—
16.3	—	—	—	—	—	—	—	0.061	—	—	—	—	—	—	—	—	—	—	—	—	—
17	—	0.056	0.111	—	—	0.106	0.010	0.081	—	0.081	0.066	—	0.222	—	—	0.020	—	—	0.061	—	—
17.2	—	—	—	—	—	—	0.005	—	—	—	—	—	—	—	—	—	—	—	—	—	—
17.3	—	—	0.025	—	—	—	—	0.040	—	—	—	—	—	—	—	—	—	—	—	—	—
18	—	0.010	0.182	—	—	0.061	—	0.000	—	0.005	0.162	—	0.141	—	—	—	—	0.020	0.076	—	—
18.3	—	—	0.030	—	—	—	—	0.025	—	—	—	—	—	—	—	—	—	—	—	—	—
19	—	—	0.141	—	—	0.045	—	—	—	—	0.162	—	0.015	—	—	—	—	0.056	0.106	—	—
19.2	—	—	—	—	—	—	—	—	—	—	—	—	—	—	—	—	—	—	0.000	—	—
19.3	—	—	0.000	—	—	—	—	0.005	—	—	—	—	—	—	—	—	—	—	—	—	—
20	—	—	0.106	—	—	0.010	—	—	—	—	0.152	—	0.000	—	—	—	—	0.141	0.086	—	—
20.2	—	—	—	—	—	—	—	—	—	—	—	—	—	—	—	—	—	—	0.020	—	—
21	—	—	0.131	—	—	0.020	—	—	—	—	0.020	—	—	—	—	—	—	0.141	0.020	—	—
21.1	—	—	—	—	—	—	—	—	—	—	—	—	—	—	—	—	—	—	0.000	—	—
21.2	—	—	—	—	—	—	—	—	—	—	—	—	—	—	—	—	—	—	0.005	—	—
21.3	—	—	—	—	—	—	—	—	—	—	—	—	—	—	—	—	—	0.000	—	—	—
22	—	—	0.116	—	—	—	—	—	—	—	0.056	—	—	—	—	—	—	0.126	0.015	—	0.005
22.2	—	—	0.005	—	—	—	—	—	—	—	—	—	—	—	—	—	—	0.010	0.010	—	—
23	—	—	0.101	—	—	—	—	—	—	—	0.162	—	—	—	—	—	—	0.202	0.000	—	—
23.2	—	—	—	—	—	—	—	—	—	—	—	—	—	—	—	—	—	0.010	0.025	—	—
24	—	—	0.025	—	—	—	—	—	—	—	0.066	—	—	—	—	—	—	0.157	0.000	—	—
24.2	—	—	—	—	—	—	—	—	—	—	—	—	—	—	—	—	—	0.000	0.010	—	—
25	—	—	0.005	—	—	—	—	—	—	—	0.136	—	—	—	—	—	—	0.081	—	—	—
25.2	—	—	—	—	—	—	—	—	—	—	—	—	—	—	—	—	—	—	0.035	—	—
26	—	—	—	—	—	—	—	—	—	—	0.010	—	—	—	—	—	—	0.040	—	—	—
26.2	—	—	—	—	—	—	—	—	—	—	—	—	—	—	—	—	—	—	0.066	—	—
27	—	—	—	—	—	—	—	—	0.010	—	—	—	—	—	—	—	—	0.010	—	—	—
27.2	—	—	—	—	—	—	—	—	—	—	—	—	—	—	—	—	—	—	0.045	—	—
28	—	—	—	—	—	—	—	—	0.207	—	0.005	—	—	—	—	—	—	0.005	—	—	—
28.2	—	—	—	—	—	—	—	—	—	—	—	—	—	—	—	—	—	—	0.040	—	—
29	—	—	—	—	—	—	—	—	0.172	—	—	—	—	—	—	—	—	—	—	—	—
29.2	—	—	—	—	—	—	—	—	—	—	—	—	—	—	—	—	—	—	0.111	—	—
30	—	—	—	—	—	—	—	—	0.172	—	—	—	—	—	—	—	—	—	0.005	—	—
30.2	—	—	—	—	—	—	—	—	0.035	—	—	—	—	—	—	—	—	—	0.066	—	—
31	—	—	—	—	—	—	—	—	0.025	—	—	—	—	—	—	—	—	—	—	—	—
31.2	—	—	—	—	—	—	—	—	0.131	—	—	—	—	—	—	—	—	—	0.040	—	—
32	—	—	—	—	—	—	—	—	0.000	—	—	—	—	—	—	—	—	—	—	—	—
32.2	—	—	—	—	—	—	—	—	0.182	—	—	—	—	—	—	—	—	—	0.015	—	—
33	—	—	—	—	—	—	—	—	—	—	—	—	—	—	—	—	—	—	0.010	—	—
33.2	—	—	—	—	—	—	—	—	0.056	—	—	—	—	—	—	—	—	—	0.000	—	—
34.2	—	—	—	—	—	—	—	—	0.000	—	—	—	—	—	—	—	—	—	0.005	—	—
35.2	—	—	—	—	—	—	—	—	—	—	—	—	—	—	—	—	—	—	0.005	—	—

**Table 4:** Different forensic parameters in the Brahmin population of Rajasthan

Locus	PIC	PM	PD	PE	TPI	Na	Ne	Ho	He	uHe
CSF1PO	0.655	0.150	0.850	0.505	1.980	6.000	3.397	0.747	0.706	0.709
D10S1248	0.737	0.089	0.911	0.540	2.152	8.000	4.379	0.768	0.772	0.776
D12S391	0.868	0.033	0.967	0.752	4.125	15.000	8.334	0.879	0.880	0.884
D13S317	0.742	0.089	0.911	0.488	1.904	8.000	4.422	0.737	0.774	0.778
D16S539	0.769	0.088	0.912	0.558	2.250	7.000	4.885	0.778	0.795	0.799
D18S51	0.849	0.043	0.957	0.814	5.500	11.000	7.331	0.909	0.864	0.868
D19S433	0.772	0.065	0.935	0.488	1.904	13.000	4.859	0.737	0.794	0.798
D1S1656	0.886	0.029	0.971	0.712	3.536	15.000	9.571	0.859	0.896	0.900
D21S11	0.823	0.051	0.949	0.712	3.536	10.000	6.356	0.859	0.843	0.847
D22S1045	0.675	0.121	0.879	0.540	2.152	9.000	3.462	0.768	0.711	0.715
D2S1338	0.853	0.041	0.959	0.793	4.950	12.000	7.565	0.899	0.868	0.872
D2S441	0.710	0.119	0.881	0.633	2.750	10.000	3.913	0.818	0.744	0.748
D3S1358	0.719	0.102	0.898	0.558	2.250	6.000	4.157	0.778	0.759	0.763
D5S818	0.675	0.125	0.875	0.455	1.768	6.000	3.609	0.717	0.723	0.727
D7S820	0.778	0.068	0.932	0.558	2.250	8.000	5.171	0.778	0.807	0.811
D8S1179	0.827	0.052	0.948	0.732	3.808	11.000	6.556	0.869	0.847	0.852
DYS391	0.370	0.565	0.435	0.000	0.500	4.000	1.771	0.000	0.435	0.437
FGA	0.852	0.038	0.962	0.793	4.950	13.000	7.505	0.899	0.867	0.871
SE33	0.935	0.018	0.982	0.752	4.125	29.000	16.160	0.879	0.938	0.943
TH01	0.762	0.077	0.923	0.540	2.152	6.000	4.853	0.768	0.794	0.798
TPOX	0.648	0.145	0.855	0.408	1.597	6.000	3.312	0.687	0.698	0.702
vWA	0.783	0.065	0.935	0.672	3.094	9.000	5.230	0.838	0.809	0.813
Mean		1.02E–25	1.000	0.999	1.07E+09	10.091	5.764	0.771	0.787	0.791
SE						1.114	0.640	0.039	0.022	0.022

**Figure 1.**
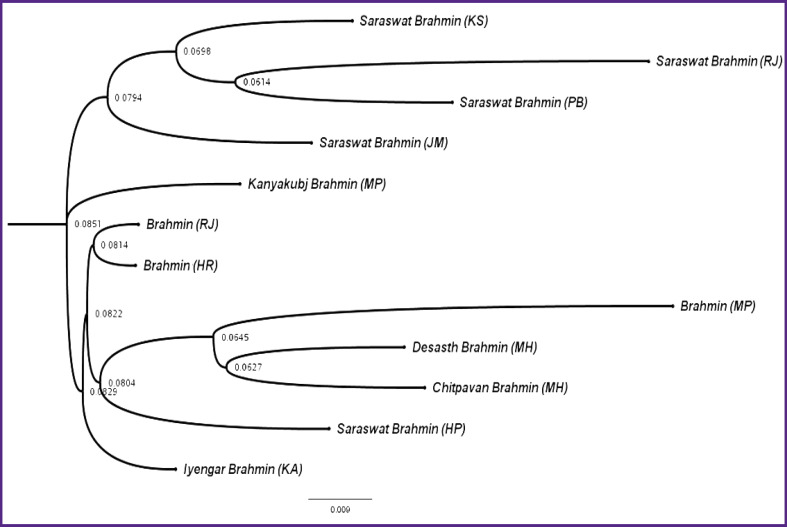
UPGMA tree showing the genetic relationship of the Brahmin population of Rajasthan and Haryana with other reported Brahmin populations residing in India

These results imply that 21 autosomal STR loci were more polymorphic to a greater extent and might be utilized for individuals’ forensic identification and parentage testing. The genetic affinity of the examined population, i.e. Brahmin of Rajasthan (RJ) and Brahmin of Haryana (HR), was compared to the previously reported Indian Brahmin population, namely, Saraswat Brahmin of Kashmir (KS), Rajasthan (RJ), Punjab (PB), Jammu (JM) and Himachal Pradesh (HP) [17], Kanyakubj Brahmin of Madhya Pradesh (MP) [18], Brahmin (MP) [19], Desasth Brahmin of Maharashtra (MH), Chitpavan Brahmin (MH) [20], and Iyengar Brahmin of Karnataka (KA) [21] populations, using the UPGMA dendrogram and POPTREE2 software. The UPGMA dendrogram based on D_sw_ genetic distance revealed that the Brahmin community shared a genetic affinity with Himachal Pradesh’s Saraswat Brahmin (Figure 1).

**Figure 2.**
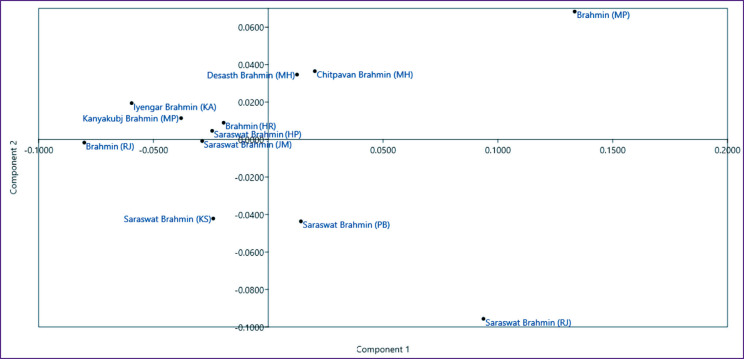
PCA plot of Brahmin population of Rajasthan and Haryana showing genetic distance relationship with other reported Brahmin populations residing in India

The PCA result of 10 populations was found to be consistent with the UPGMA dendrogram, indicative of the genetic relatedness of the Brahmin population of Haryana and Rajasthan with other Indian populations (Figure 2).

## Conclusion

This has been a pilot study to bring the genetic characteristics of the Haryana and Rajasthan Brahmin population together and demonstrating the utility of a panel of specific markers in the disputed forensic cases. The genetic data from the present study could be helpful in population genetics and forensics. We suggest further extending this study encompassing a greater more number of samples. It can be conducted by using the representative samples belonging to varied castes/ ethnic groups while keeping the immigration routes of the human population in Haryana and Rajasthan states under consideration. In addition, the fine scale understanding of population assignment can be aided by application of genomic data using next generation sequencing. In addition, based on the DNA technology, Regulation bill was enacted by the Ministry of Science and Technology of the Indian Government in Lok Sabha (2019). The presently conducted study would aid in the development of DNA Data Banks at national, as well as state, levels.
